# Extracellular Superoxide Dismutase (EC-SOD) Regulates Gene Methylation and Cardiac Fibrosis During Chronic Hypoxic Stress

**DOI:** 10.3389/fcvm.2021.669975

**Published:** 2021-05-31

**Authors:** Ayan Rajgarhia, Kameshwar R. Ayasolla, Nahla Zaghloul, Jorge M. Lopez Da Re, Edmund J. Miller, Mohamed Ahmed

**Affiliations:** ^1^School of Medicine, Children's Mercy Hospital and University of Missouri-Kansas City, Kansas City, MO, United States; ^2^Henry Ford Health System, Detroit, MI, United States; ^3^Neonatal Division, University of Arizona, Tucson, AZ, United States; ^4^Neonatal Division, Orlando, Nemours Children's Hospital, Orlando, FL, United States; ^5^RDS2 Solutions, Stony Brook, New York, NY, United States

**Keywords:** hypxoia, cardiac fibrosis, EC-SOD, methylation, RASSF1A

## Abstract

Chronic hypoxic stress induces epigenetic modifications mainly DNA methylation in cardiac fibroblasts, inactivating tumor suppressor genes (RASSF1A) and activating kinases (ERK1/2) leading to fibroblast proliferation and cardiac fibrosis. The Ras/ERK signaling pathway is an intracellular signal transduction critically involved in fibroblast proliferation. RASSF1A functions through its effect on downstream ERK1/2. The antioxidant enzyme, extracellular superoxide dismutase (EC-SOD), decreases oxidative stress from chronic hypoxia, but its effects on these epigenetic changes have not been fully explored. To test our hypothesis, we used an *in-vitro* model: wild-type C57B6 male mice (WT) and transgenic males with an extra copy of human hEC-SOD (TG). The studied animals were housed in hypoxia (10% O_2_) for 21 days. The right ventricular tissue was studied for cardiac fibrosis markers using RT-PCR and Western blot analyses. Primary C57BL6 mouse cardiac fibroblast tissue culture was used to study the *in-vitro* model, the downstream effects of RASSF-1 expression and methylation, and its relation to ERK1/2. Our findings showed a significant increase in cardiac fibrosis markers: Collagen 1, alpha smooth muscle actin (ASMA), and SNAIL, in the WT hypoxic animals as compared to the TG hypoxic group (*p* < 0.05). The expression of DNA methylation enzymes (DNMT 1&3b) was significantly increased in the WT hypoxic mice as compared to the hypoxic TG mice (*p* < 0.001). RASSF1A expression was significantly lower and ERK1/2 was significantly higher in hypoxia WT compared to the hypoxic TG group (*p* < 0.05). Use of SiRNA to block RASSF1A gene expression in murine cardiac fibroblast tissue culture led to increased fibroblast proliferation (*p* < 0.05). Methylation of the RASSF1A promoter region was significantly reduced in the TG hypoxic group compared to the WT hypoxic group (0.59 vs. 0.75, respectively). Based on our findings, we can speculate that EC-SOD significantly attenuates RASSF1A gene methylation and can alleviate cardiac fibrosis induced by hypoxia.

## Introduction

Cardiac fibrosis can develop following a variety of stimuli, including ischemia, volume overload, pressure overload, and hypoxia (1). A common feature of all these stimuli is the reduced availability of oxygen. Whether from decreased oxygen delivery or from increased oxygen consumption, tissue hypoxia is associated with infiltration of inflammatory cells and activation of resident cells (2). Cardiac fibroblasts, the main resident cells, are activated and transform to myofibroblasts, which are the key driver for the fibrotic response. Other cell types act indirectly by secreting fibrogenic mediators (macrophages, mast cells, lymphocytes, cardiomyocytes, and vascular cells). Oxidative stress regulates collagen synthesis and matrix metalloproteinase activity in cardiac fibroblasts. Oxidative stress activates mitogen-activated protein kinases and stress-responsive protein kinases (3). Markers of cardiac fibrosis include collagen I and III, alpha smooth muscle actin (ASMA), and SNAIL (4). Cardiac fibrosis leads to both systolic and diastolic dysfunction and increases cell death and damage by inflammatory mediators. Prognosis depends on the etiology and extent of the disease, with some cases caused by chronic hypoxia showing some reversibility of fibrosis (4).

DNA methylation is an epigenetic modification, which plays an important role in the cellular response to chronic hypoxia and the progression of cardiac fibrosis (2). DNA methylation alters the chromatin structure leading to repression of gene expression. The methylation process is regulated by a family of DNA methyltransferase (DNMT) enzymes. Studies have demonstrated significant increases in the activities of DNMT1 (the enzyme responsible for maintaining the methylation status of daughter cells during cell cycle) and DNMT 3B (*de novo* methylating enzyme) in response to hypoxia (2). The hypoxia-induced expression of DNMT1 and DNMT 3B is in part regulated by hypoxia-inducible transcription factor 1α (HIF-1α), through specific hypoxic response elements present in the promoter sequence of DNMT1 and 3B (2). Their increased activity correlates positively with the degree of cardiac fibrosis (2). This suggests the role for epigenetic modification in fibrosis secondary to hypoxia (2).

Ras association domain family 1 isoform A (RASSF1A) is a tumor suppressor gene, and alterations in its regulation are frequently seen in cardiac fibrosis (2). RASSF1A inhibits proliferation by inhibiting the accumulation of cyclin D1 and arresting cell division and promotes apoptosis (5).

RASSF1A functions through its effect on downstream proteins such as extracellular signal-regulated kinases (ERK1/2). The Ras/ERK signaling pathway has been recognized as an intracellular signal transduction critically involved in fibroblast proliferation. Ras/ERK1/2 is activated in cardiac fibroblasts by platelet-derived growth factor-BB (PDGF-BB) and promotes cellular proliferation (6). In cardiac myocytes, RASSF1A can prevent hypertrophy through disruption of Ras/Raf-1/ERK MAPK signaling. RASSF1A also activates Mst1 to elicit apoptosis. In cardiac fibroblasts, RASSF1A represses NF-κB transcriptional activity and inhibits TNF-α production and secretion, thereby preventing paracrine-mediated hypertrophic signaling between fibroblasts and myocytes. This mechanism involves multiple cell types and paracrine signaling including calcineurin/NFAT, HDAC/MEF2, and MEK/ERK pathways, which have been elucidated in cardiac myocytes (7).

DNA methylation-mediated silencing of RASSF1A, and subsequent activation of ERK1/2, can lead to activation of fibroblasts and fibroblast proliferation (6). Another contributing mechanism to cardiac dysfunction induced by hypoxia is myofilament modification. Two myosin heavy chain (MHC) isoforms, MHC-α and MHC-β, are expressed in the mouse heart. α-MHC has higher intrinsic adenosine triphosphatase (ATPase) activity and hence contributes to higher contractility, while β-MHC has lower intrinsic ATPase activity and has a greater economy of force maintenance (8). Hypoxia has been shown to cause switching of myosin heavy chains (MHC) from its alpha to beta isoform, thereby decreasing ATPase activity and overall force of contraction (4).

Hypoxia and reactive oxygen species (ROS) play a pivotal role both in the pathogenesis of hypoxia-induced pulmonary hypertension and in the development of cardiac fibrosis (9–13). The role of ROS, as a trigger of DNA methylation of tumor suppressor gene promoters in carcinogenesis, was shown in previous studies. In human breast cancer, it increases redox concentration (e.g., hydrogen peroxide), induces the overexpression of epigenetic modifiers, including DNMT1 and HDAC1, inhibits gene expression, including tumor suppression; and enhances the expression of epithelial to mesenchymal transition inducer genes, including Snail and Slug (5, 14).

The inflammatory response mediated by extracellular reactive oxygen generated from repetitive ischemia/reperfusion in a murine model plays a critical role in the pathogenesis of fibrotic remodeling and ventricular dysfunction (7). Supplementation with vitamins E, C, and A have provided antioxidant protection against cardiomyocyte death and have improved survival in congestive heart failure models and doxorubicin-induced injury (11). Doxorubicin causes cardiotoxicity through the generation of ROS (11). ROS generated by doxorubicin include superoxide, hydrogen peroxide, hydroxyl radical, peroxynitrite, and lipid hydroperoxide, which are messengers for signaling apoptotic cell death (11). *In-vitro* cardiomyocyte studies suggest that superoxide dismutase-like and glutathione peroxidase-like compounds can protect against free radical production and cellular apoptosis due to doxorubicin (6). EC-SOD is an important antioxidant throughout the cardiovascular system and has been shown to protect the heart from ischemic damage, hypertrophy, and inflammation (15–17). Studies show that human populations with a mutation in the matrix-binding domain of EC-SOD, which diminishes its affinity for the extracellular matrix, have a higher risk for the development of cardiovascular and ischemic heart disease (4). EC-SOD is important in the prevention of oxidative injury that may contribute to cardiac remodeling, in myocardial infarction models, and alter *ex vivo* heart function (16, 17). EC-SOD overexpression has also been shown to decrease the fibrosis that develops in cardiac tissue secondary to ischemia–reperfusion injury (10, 18). However, the specific mechanisms by which EC-SOD protects against fibrosis and tissue damage, in various organs including the lung and heart, remain unclear. Also, the relation between EC-SOD and epigenetic changes has not been fully explored. In this study, we reveal the effects of overexpression of EC-SOD on cardiac fibrosis, epigenetic changes, and myofilament changes due to chronic hypoxic stress.

## Materials and Methods

### *In-vivo* Studies

All experiments involving animals were reviewed and approved by the Institutional Animal Care and Use Committee of the Feinstein Institute for Medical Research. Adult (8–10-week-old) C57BL6 male mice (wild type—WT) and transgenic neonate mice, with an extra copy of the human EC-SOD gene containing a β-actin promoter (TG), were housed in a pathogen-free environment under standard light and dark cycles, with free access to food and water (19). hEC-SOD TG mice with C57BL6 background were studied before, by us and other researchers, and have been well-characterized (19, 20). TG mice act and behave similarly under normal conditions (room air), like WT mouse strains as shown in many studies before (19, 20).

### Animals

The studied animals were divided into three groups, Group A: WT mice housed in room air; Group B: WT mice housed in 10% normobaric oxygen for 21 days using a BioSpherix chamber (Lacona, NY, USA) (WT hypoxic group); and Group C: TG mice housed under the same hypoxic conditions as Group B (18). After 21 days, the animals were euthanized under a surgical plane of anesthesia [Fentanyl/Xylazine (5:1)] (21), and right ventricular tissue was harvested for analysis.

#### RT-qPCR

Gene expression, within the right ventricular tissue samples, was assessed following tissue disruption and homogenization. RNA was then extracted from the tissue using the AllPrep DNA/RNA extraction kit (Qiagen), according to the manufacturer's instructions. First-strand cDNA synthesis was carried out using SuperScript II RT (Invitrogen). Quantitative real-time PCR primers were designed so that one of each primer pair was exon/exon boundary spanning to ensure that only mature mRNA was amplified. The sequences of the gene-specific primers used were ASMA, 5′-aatgagcgtttccgttgc 3′ (forward), 5′-atccccgcagactccatac-3′ (reverse); collagen 1a 1 (COL1A1), 5′-catgttcagctttgtggacct 3′ (forward), 5′ gcagctgacttcagggatgt 3′ (reverse); collagen 3 a 1 (COL 3A1), 5′ tcccctggaatctgtgaatc 3′ (forward), 5′ tgagtcgaattggggagaat 3′ (reverse); α-Myosin Hea vy Chain (Myh6) 5′ cgcatcaaggagctcacc-3′ (forward), 5′-cctgcagccgcattaagt-3′ (reverse); and β-Myosin Heavy Chain (Myh7) 5′-cgcatcaaggagctcacc-3′ (forward), 5′-ctgcagccgcagtaggtt-3′ (reverse). Q-PCR was performed; amplification and detection were carried out using Roche Applied Science LightCycler 480 PCR Systems with software. The PCR cycling program consisted of 45 three-step cycles of 15 s/95°C, 1 min/57°C, 1 s/72°C. Relative changes in mRNA expression were calculated as fold changes (normalized using *Gapdh*) by using the comparative Ct (ΔΔCt) method (22).

#### Immunohistochemistry—Collagen 1 (Cy 3)

Right ventricular tissue, fixed in 4% paraformaldehyde for 24 h, was processed, embedded in paraffin, and subsequently cut into 4-μm-thick sections. The slides then underwent heat-mediated antigen retrieval followed by incubation with primary antibody anti-Collagen 1 antibody-3G3 (Abcam ab88147) and secondary antibody AffiniPure Goat Anti-Mouse IgG (Jackson ImmunoResearch Lab Inc, Code 115005003). The slides were then analyzed using an Olympus FluoView FV300 Confocal Laser Scanning Microscope (Thermo Fisher Scientific), and the Fiji image processing software (an open-source platform for biological image analysis) was used for analysis of pixel density (23).

#### Western Blot Analysis

Frozen right ventricular tissues were homogenized, and protein extraction was carried out using a total protein extraction Kit (BioChain Institute, Inc. Hayward, CA). The protein concentration was evaluated using the Modified Lowry Protein Assay (Thermo Fisher Scientific, Rockford, IL, USA). Samples were prepared for SDS-PAGE in Laemmli sample buffer (Bio-Rad, Hercules, CA, USA) and processed as previously described (23). Membranes were briefly washed and immediately incubated with the respective primary antibody in 5% BSA with phosphate-buffered saline with Tween 20 (PBST), overnight. Following washing with PBST, the membranes were incubated with horseradish peroxidase-conjugated secondary antibodies for 40–60 min. The membranes were washed and then processed using Amersham ECL detection systems (GE Healthcare, Piscataway, NJ, USA). The membranes were immediately exposed to 8610 Fuji X-Ray Film. The films were assessed using Quantity One 1-D Analysis Software on a GS-800 Calibrated Densitometer. The density of each band is presented as a ratio in comparison to the Actin band density. Primary antibodies were used to detect the following markers: Collagen 1, Alpha Smooth Muscle Actin, SNAIL, DNMT1 and 3b, and HIF1α (each obtained from Abcam, Cambridge, MA); RASSF1A (Origene, Rockville, MD); and ERK 1/2 (Cell Signaling Technology, Danvers, MA, USA).

#### ROS Assay

Intracellular ROS was assessed using a cell-based assay for measuring hydroxyl, peroxyl, or other reactive oxygen species. The assay employs the cell-permeable fluorogenic probe 2′,7′-dichlorodihydrofluorescein diacetate (DCFH-DA) (Cell Biolabs, San Diego, CA).

### *In-vitro* Analysis

Primary C57BL6 Mouse Cardiac Fibroblasts (MCF) (Cell Biologics, Chicago, IL, Catalog No. C57-6049) were grown to confluence in T75 flasks using the fibroblast growth medium (Cell Biologics, Chicago, IL, Catalog No. M2267), in a humidified incubator (37°C, 5% CO_2_). Cells were seeded to six-well tissue culture plates, at a density of 10,000 cells per well, and incubated for 24 h. Next, the cells were transfected with human EC-SOD (hEC-SOD) cDNA inserted into a vector plasmid pcDNA3 (5446 nucleotides; Invitrogen Life Technologies, Carlsbad, CA, USA) as previously described (13), using the FuGENE kit (Roche Diagnostics, Indianapolis, IN, USA). Each well received 1 μg DNA/100 μL serum-free medium of the DNA/FuGENE complex. Control wells received the serum-free medium alone. Transfected cells were selected using Geneticin (Invitrogen Life Technologies). Transfection of the fibroblasts was confirmed by Western blot analysis using an antibody specific for hEC-SOD (R&D Systems, Minneapolis, MN, US).

#### Quantitative Flow Cytometry—Effects of Hypoxia on Global DNA Methylation Profile

A modular incubator chamber (Billups-Rothenberg, Del Mar, CA, USA) was used for the cell hypoxia studies and a 1% oxygen atmosphere maintained using an oxygen sensor (BioSpherix, Lacona, NY, USA). Cells were maintained in a microenvironment of 37°C, 1% O_2_, 5% CO_2_, and 100% humidity. To examine the effects of hypoxia ± EC-SOD overexpression on global DNA methylation, both transfected and non-transfected MCF were incubated in hypoxia for 72 h. Control, non-transfected MCF were maintained in 21% oxygen for 72 h. Post culture, MCF cells were fixed in Carnoy's solution prior to 60-min acid hydrolysis in 1 M HCl at 37°C. Following this DNA denaturation step, cells were then treated with either an anti-5′ methylcytidine (5MeC) monoclonal antibody (EpiGentek, Catalog No. A-1014) or a non-specific IgG1 antibody (BD Biosciences, San Jose, CA). IgG1-negative controls were used at the same concentration as the primary antibody. Immunostaining was conducted using an FITC-conjugated rabbit anti-mouse secondary antibody (Thermo Scientific, Catalog No. 31561). The cells were then subjected to flow cytometry (BD Biosciences, San Jose, CA) and the results assessed using CellQuest Pro (BD Biosciences, San Jose, CA).

#### Proliferation Studies—Effects of Blocking RASSF1A Expression

The expression of RASSF1A was reduced by transfection of MCF with small interfering RNA (SiRNA) SiRASSF1A (Thermo Fisher Scientific, catalog no. 185488) located on Chr.9: 107551555–107562267 on Build GRCm38MCF, using Lipofectamine RNAiMAX transfection protocol (Life Technologies). Effectiveness of the transfection was evaluated at 24, 48, 72, and 96 h post transfection with western blot analysis using an antibody specific for RASSF1A (Abcam). Post-transfection studies showed that RASSF1A expression is minimal after 72 h of transfection (data are not shown). This time point was used for cell proliferation assessment in the next step. Cells were housed in a humidified incubator (37°C, 5% CO_2_) and compared to control MCF, which were transfected with an empty vector and kept under the same conditions. After 72 h of transfection, MCF proliferation was assessed by BrdU (5-bromouridine) incorporation, (Roche Diagnostics, Mannheim). Cell counts were performed using a hemocytometer 24 and 48 h later.

#### Methylation Study

Bisulfite chemically converts unmethylated cytosine to uracil but has no effect on methylated cytosine. To determine if promoter region hypermethylation could be responsible for the downregulation of RASSF1A expression, direct bisulfite sequencing on mouse cardiac fibroblasts was performed (17). The methylation ratio (meth-ratio) was calculated using methylated CpG total CpG counts.

### RASSF1A Methylation Analysis

Briefly, the genomic DNA from mouse heart was isolated using the TRIzol solution (Invitrogen, Thermo Fisher Scientific Inc), according to the manufacturer's protocols. The methylation status of the RASSF1A promoter region was determined by chemical modification of genomic DNA with sodium bisulfite and methylation-specific PCR. The bisulfite-treated DNA was used as a template for the methylation-specific PCR reaction. Primers for the unmethylated DNA-specific reaction were F, 5V-GGTGTTGAAGTTGTGGTTTG-3V; R, 5V-TATTATACCCAAAACAATACAC-3V. Primers for the methylated DNA-specific reaction were F, 5V-TTTTGCGGTTTCGTTCGTTC-3V; R, 5V-CCCGAAACGTACTACTATAAC-3V. The reactions were incubated at 95°C for 1 min, 55°C for 1 min, and 72°C for 1 min, for 35 cycles. The amplified fragment was confirmed by DNA sequencing. DNA from normal heart was used as a control for unmethylated RASSF1A. The strategies for RASSF1A sequencing and the amplicons map Supplementary #1, #2, respectively.

### Statistics

Data was expressed as the mean ± standard error of the mean (SEM). Unless otherwise indicated, a one-way or two-way analysis of variance followed by Bonferroni *post-hoc* test was used to assess significance (*P* < 0.05) using GraphPad Prism 8 (GraphPad Software, Inc, La Jolla, CA).

## Results

A comparison between WT adult mice and TG mice housed in room air was performed and is shown in Supplementary #3. All molecular testing showed no significant difference between WT and TG under normoxic conditions (room air).

### EC-SOD Reduces Cardiac Fibrosis

**Figure 1:**
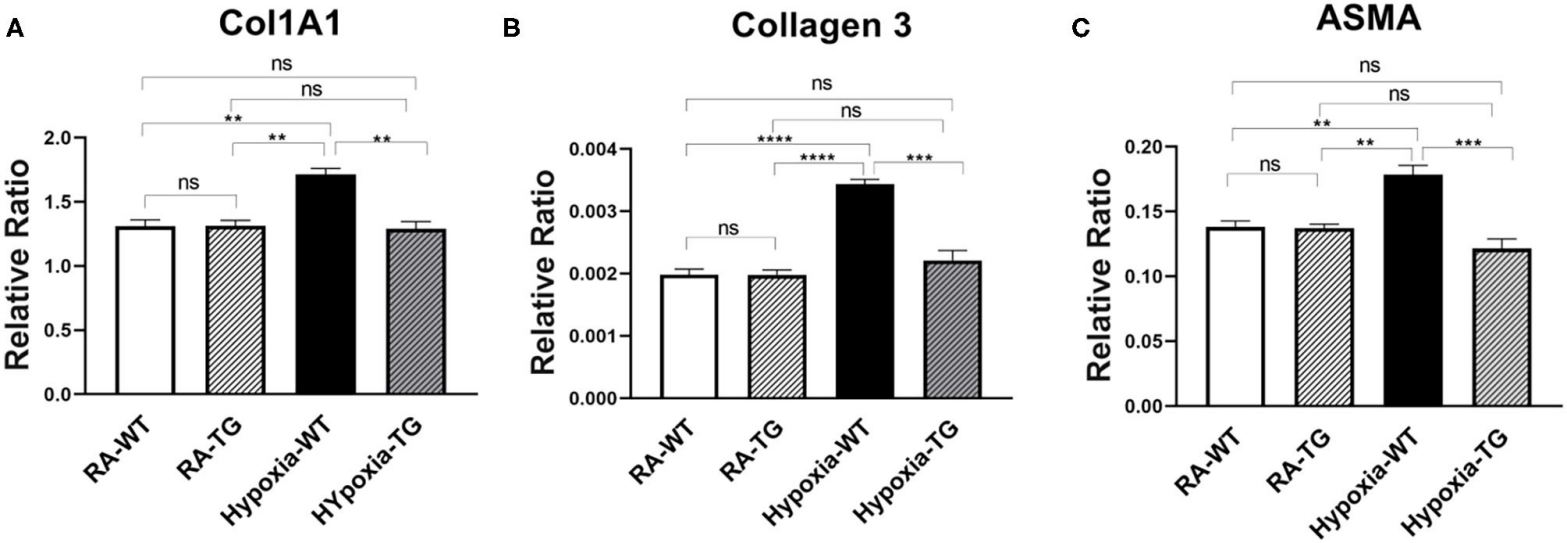
RT-PCR for markers of fibrosis—Collagen 1, Collagen 3, and ASMA. Adult male C57B6 mice, WT, and EC-SOD transgenic mice (TG) were exposed to FiO_2_ 10% hypoxia for 21 days (WT). The control group was composed of animals housed in room air (RA). Quantitative RT-PCR was used to assess gene expression analysis of Collagen 1 **(A)**, Collagen 3 **(B)**, and ASMA **(C)** in the right ventricular tissue. All experiments *n* = 5. Data represents mean ± SEM. *P < 0.05, **P < 0.01, ***P < 0.001, ****P < 0.0001.

To examine the effects of EC-SOD overexpression on markers of cardiac fibrosis at the mRNA level, we performed gene expression analysis using RT-PCR. There was a significant decrease in the expression of the cardiac fibrosis markers, Collagen 1 (Col1A1) (Figure 1A), Collagen 3 (Figure 1B), and ASMA (Figure 1C) in the hypoxic TG, as compared to the hypoxic WT animals (*p* < 0.05). The gene expression of Collagen 1, Collagen 3, and ASMA in hypoxic TG group was not statistically significantly different from room air controls. These results show that EC-SOD overexpression reduces gene expression of cardiac fibrosis markers to levels comparable to RA groups.

**Figure 2:**
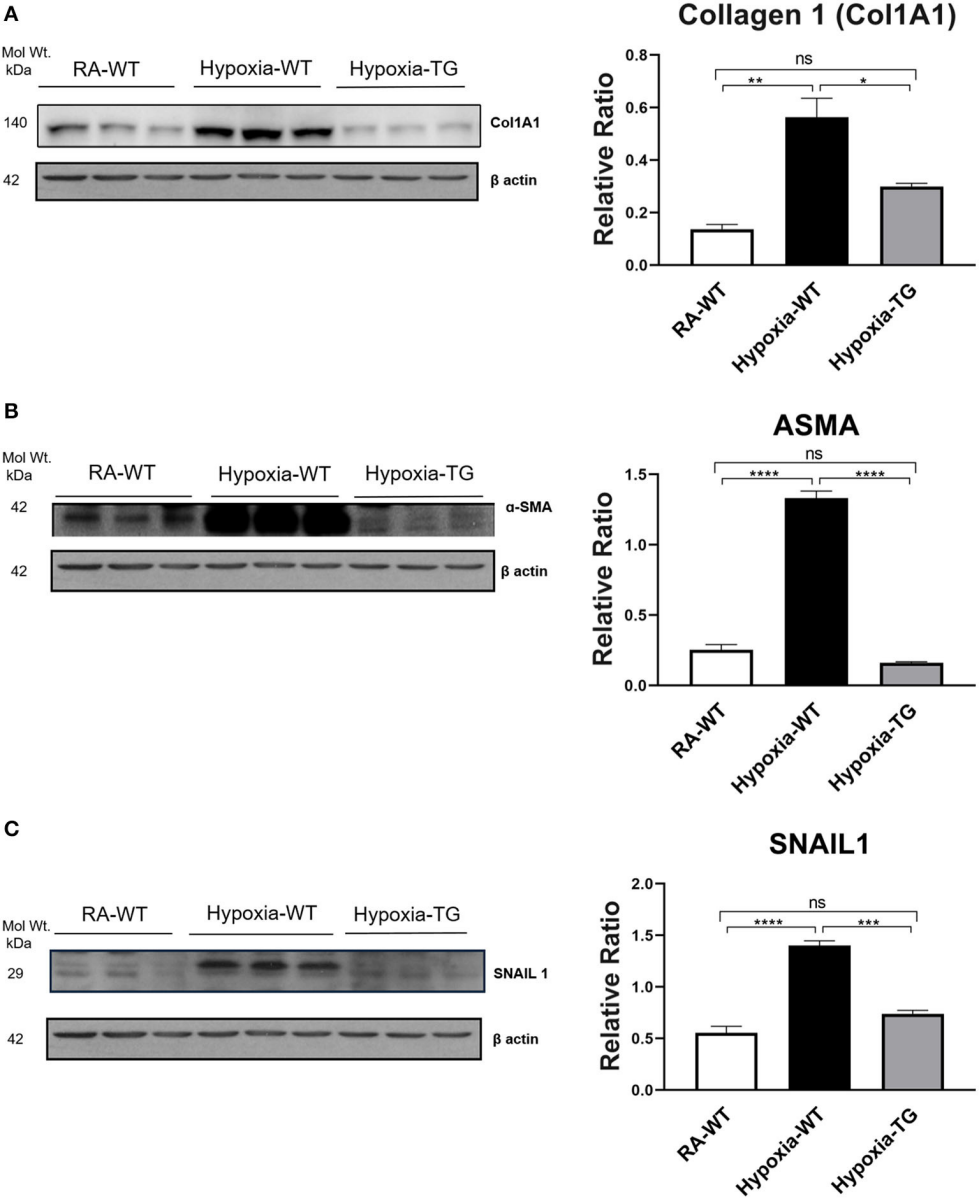
Western blot analysis of markers of fibrosis—Collagen 1, ASMA, and SNAIL. Adult mice (C57B6) (WT) and EC-SOD transgenic mice (TG) were exposed to FiO_2_ 10% hypoxia for 21 days (WT). Room air animals were used as control group (RA). WB analysis was used to assess protein levels of Collagen 1 **(A)**, ASMA **(B)**, and SNAIL **(C)** in the right ventricular tissue. All experiments *n* = 3. Data represents mean ± SEM. *P < 0.05, **P < 0.01, ***P < 0.001, ****P < 0.0001.

**Figure 3:**
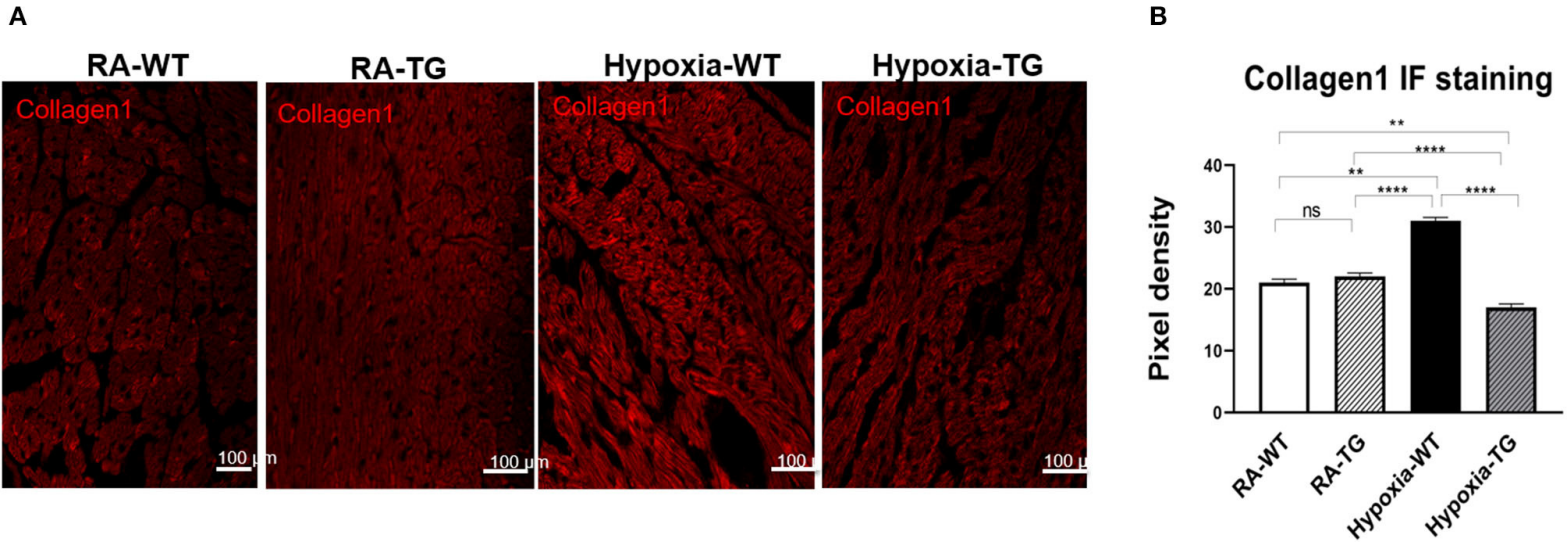
Immunohistochemistry for Collagen 1. Right ventricular tissue from adult wild-type mice WT and EC-SOD transgenic mice (TG), which were exposed to FiO_2_ 10% hypoxia for 21 days (WT) and a room air control group (RA) were treated with Cy3 stain to assess for levels of Collagen 1 **(A)**. The images were analyzed using Fiji image processing software to measure pixel density **(B)**. All experiments *n* = 5. Data represents mean ± SEM. *P < 0.05, **P < 0.01, ***P < 0.001, ****P < 0.0001.

To confirm that protein levels of cardiac fibrosis markers showed a similar trend to gene expression level, we performed protein-level assessment using Western blot analysis. There was a significant increase in the cardiac fibrosis marker levels of Collagen 1 (Figure 2A), ASMA (Figure 2B), and SNAIL1 (Figure 2C) in hypoxic WT animals as compared to hypoxic TG animals (*p* < 0.05). The protein concentration of these three markers in the hypoxia TG animals was not significantly different from the RA control group. This indicates that EC-SOD overexpression reduces the protein expression of cardiac fibrosis markers to levels comparable to RA groups. Immunohistochemistry staining for Collagen 1 showed a significant decrease in pixel density of Collagen 1 in the hypoxic TG animals, as compared to the hypoxic WT animals (Figures 3A,B) (*P* < 0.05).

### EC-SOD Reduces Epigenetic Modifications—DNA Methylation

**Figure 4:**
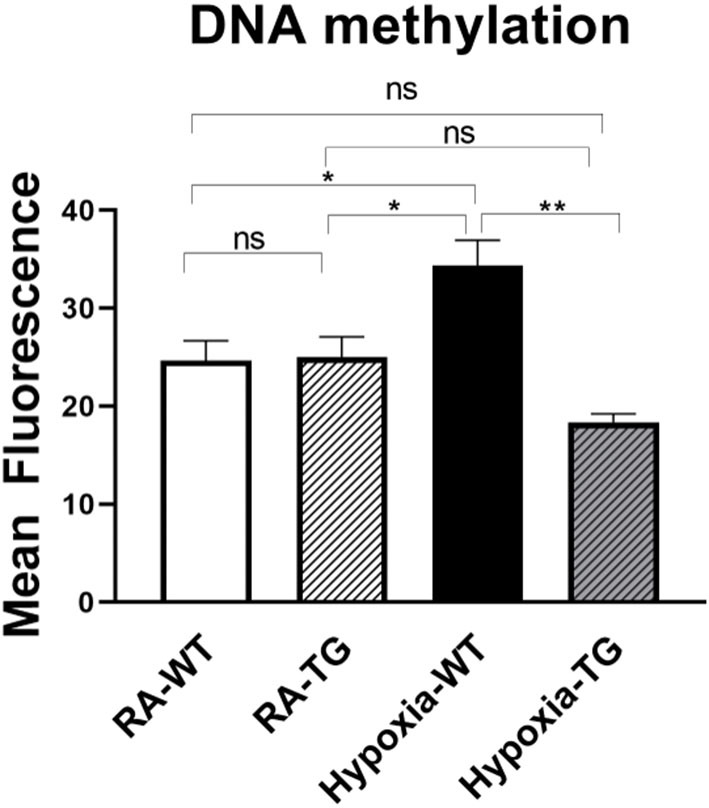
Flow cytometry studies for DNA methylation—In *in-vitro* studies using cardiac fibroblasts, DNA methylation levels were assessed in cells exposed to hypoxic conditions (FiO_2_ 1% for 72 h) and compared to cells transfected with EC-SOD and exposed to hypoxia. Cells cultured in room air were employed as controls. Triplicate wells were analyzed, and the experiments were repeated five times. Data represents mean ± SEM. *P < 0.05, **P < 0.01, ***P < 0.001, ****P < 0.0001.

To examine the effects of EC-SOD on DNA methylation, the most common form of epigenetic modifications, we transfected cardiac fibroblast with hEC-SOD and subjected them to hypoxia. Quantitative flow cytometry studies, using antibody directed to methylated DNA, revealed a significant increase in global DNA methylation in cardiac fibroblasts subjected to hypoxia, compared to cardiac fibroblasts transfected with hEC-SOD and subjected to the same hypoxic conditions (*p* < 0.05) (Figure 4), thus showing that *in-vitro*, EC-SOD significantly decreased global DNA methylation under hypoxic conditions.

**Figure 5:**
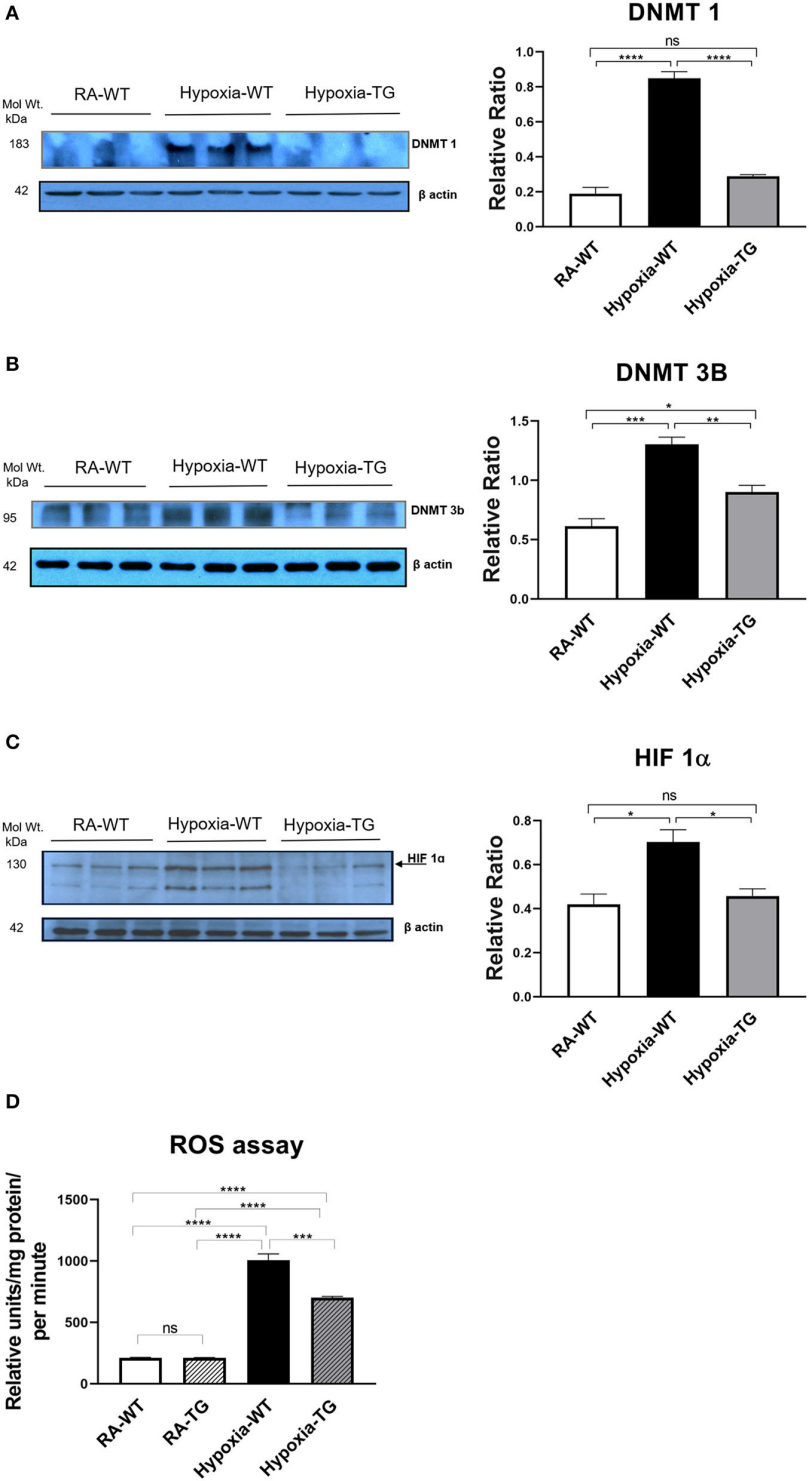
Western blot analysis for DNMT1, 3b, and HIF-1**α**. Adult mice (WT) and EC-SOD transgenic mice (TG) were exposed to FiO_2_ 10% hypoxia for 21 days. Room air animals were used as a control group (RA). WB analysis was used to assess protein levels of DNMT 1 **(A)** and DNMT 3b **(B)**; HIF-1α assessment in cardiac fibroblasts **(C)** in the right ventricular tissue. ROS assay in all studied groups **(D)**. All experiments were carried out in triplicate. Data represent mean ± SEM. *P < 0.05, **P < 0.01, ***P < 0.001, ****P < 0.0001.

To examine if the same results hold true in the *in-vivo* environment, we performed protein assessment of DNA methylating enzymes, using Western blot analysis. There was a significant decrease in the DNA methylating enzymes, DNMT1 (Figure 5A) and DNMT-3b (Figure 5B), in the hypoxic TG animals compared to the hypoxic WT animals (*p* < 0.05). The levels of HIF1α (Figure 5C) were also noted to be significantly reduced in the hypoxic TG animals when compared to the WT animals in hypoxia (*p* < 0.05). However, the levels in the hypoxic TG animals were not significantly different when compared to the TG room air group. Assay of free reactive oxygen species accumulation by DCF assay showed a significant increase of ROS in the WT hypoxic group, with ROS levels being significantly lower in the TG hypoxic group (Figure 5D). Thus, EC-SOD overexpression decreases DNA methylation, HIF1α, and ROS *in-vivo* under hypoxic conditions.

### EC-SOD Ameliorates the Hypoxia-Induced Epigenetic Modifications to RASSF1A Through the Ras/ERK Pathway

We have shown that hypoxia induces epigenetic modifications both *in-vitro* and *in-vivo*. RASSF1A is a tumor suppressor gene, frequently involved in cardiac fibrosis. DNA methylation-mediated silencing of RASSF1A leads to fibroblast proliferation and cardiac fibrosis. We wanted to examine the effects of EC-SOD overexpression on RASSF1A and further examine the Ras/ERK pathway.

**Figure 6:**
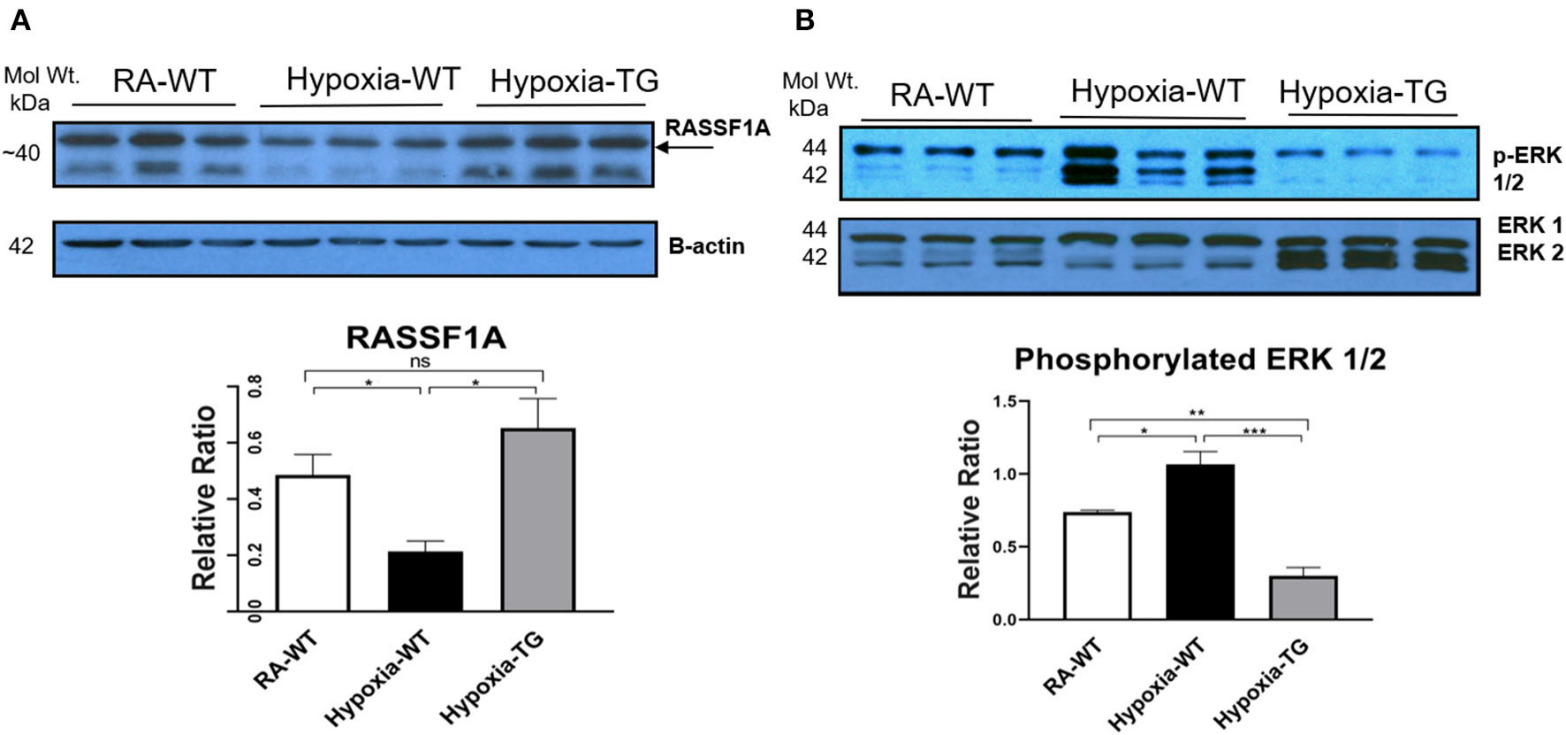
Western blot analysis for RASSF1A and ERK1/2. Adult mice (C57B6) (WT) and EC-SOD transgenic mice (TG), were exposed to 10% hypoxia for 21 days (WT). Room air animals were used as control group (RA). WB analysis was used to assess protein levels of RASSF1A **(A)** and ERK1/2 **(B)** in the right ventricular tissue. All experiments were carried out in triplicate. Data represents mean ± SEM. *P < 0.05, **P < 0.01, ***P < 0.001, ****P < 0.0001.

Western blot analysis in the hypoxic WT animals showed a significant reduction in the gene RASSF1A (Figure 6A), as compared to both hypoxic TG animals and RA control groups (*p* < 0.05). WT hypoxic animals showed a significant increase of ERK phosphorylation in comparison to the RA control group (*P* < 0.05). The level of ERK 1/2 (Figure 6B) was significantly reduced in the hypoxic TG animals compared to the hypoxic WT animals.

### EC-SOD Prevents Myofilament Changes

**Figure 7:**
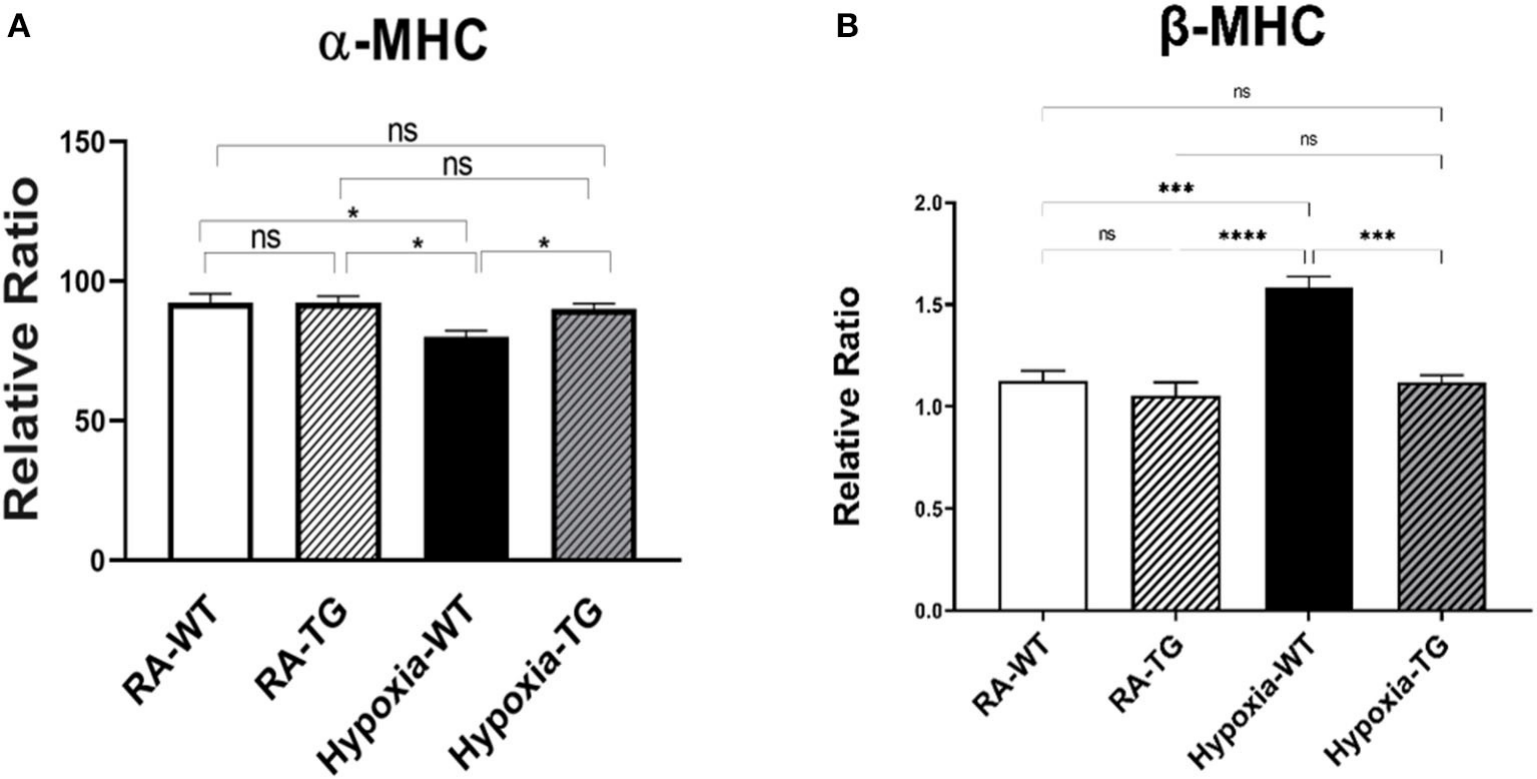
**(A,B)** RT-PCR for α and β myosin heavy chain. Adult mice (C57B6) (WT) and EC-SOD transgenic mice (TG), were exposed to 10% hypoxia for 21 days (WT). Room air animals were used as control group (RA). Quantitative RT-PCR was used to assess gene expression analysis of α-MHC in the right ventricular tissue. All experiments *n* = 5. Data represents mean ± SEM. *P < 0.05, **P < 0.01, ***P < 0.001, ****P < 0.0001.

Myofilament modification induced by hypoxia causes cardiac dysfunction. Hypoxia can cause switching of Myosin Heavy chains (MHC) from its alpha (high ATP) to beta isoform low ATP, thereby decreasing contractility (Figures 7A,B). Gene expression analysis using RT-PCR showed a significant reduction in the levels of α-MHC and a significant increase in β-HMC in hypoxic WT animals as compared to RA groups and hypoxic TG group (*P* < 0.05), and this switch was reversed among TG hypoxic group. This provides evidence of EC-SOD overexpression improving cardiac contractility.

### RASSF1A Silencing Increase Cardiac Fibroblast Proliferation

**Figure 8:**
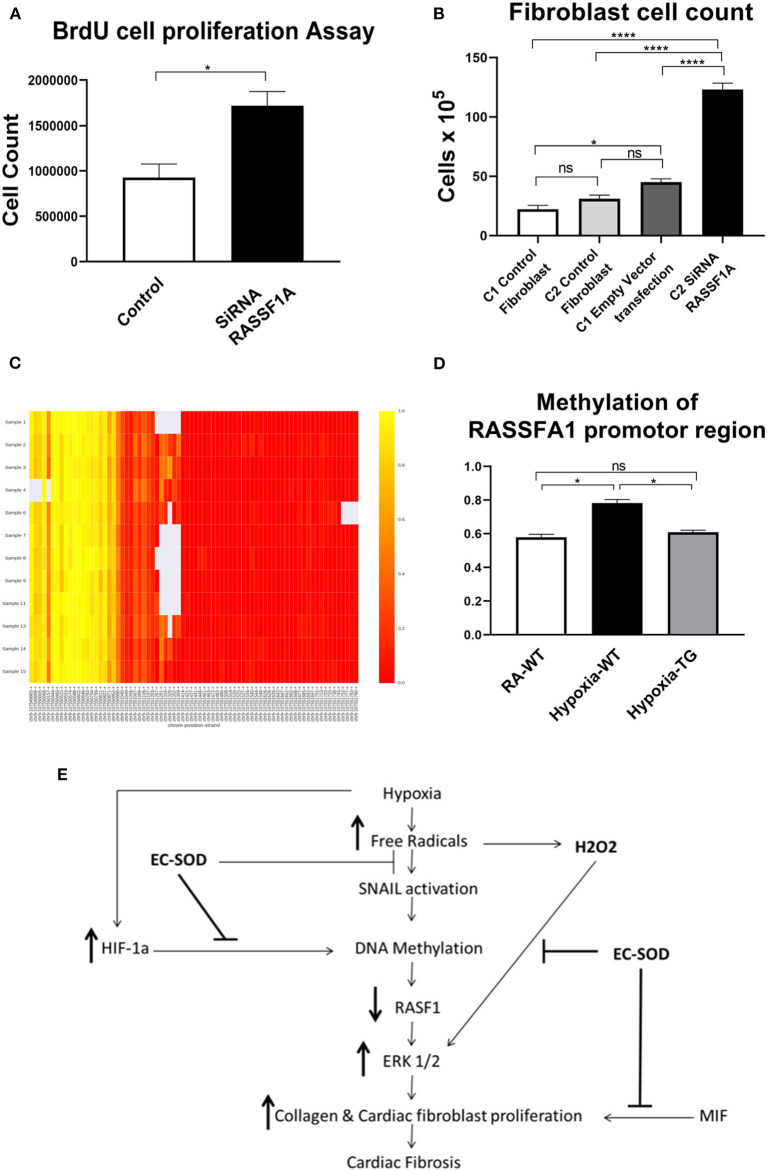
Cell proliferation studies after transfection with SiRNA for RASSF1A—Cardiac fibroblasts were transfected with SiRNA for RASSF1A and compared to cells placed in RA which served as a control. BrdU cell proliferation studies were undertaken **(A)**, and cells as having underwent cell counts **(B)**. DNA methylation studies of the RASSF1A promotor region as shown in the heat map: RA group (samples 1–4); WT hypoxic group (Samples 6–9); TG hypoxic group (samples 11–15) **(C)**. Quantitative estimation of methylation level among the three studied groups **(D)**. Proposed mechanism **(E)**. Data represent mean ± SEM. *P < 0.05, **P < 0.01, ***P < 0.001, ****P < 0.0001.

To provide further evidence that RASSF1A is involved in cardiac fibroblast proliferation, we incubated mouse cardiac fibroblast (MCF), with SiRNA blocking the expression of RASSF1A and incubated in room air condition, both BrdU assay and cell count studies were performed. BrDU analysis (Figure 8A) showed a significant increase in cell proliferation post-transfection with SiRNA, inhibiting RASSF1A expression, as compared to control cells also incubated in room air (Figure 8B). There was a significant increase in cell numbers, with SiRNA silencing RASSF1A, as compared to control cells transfected with an empty vector. These findings support that silencing RASSF1A, leads to activation of ERK1/2, which stimulate cell proliferation.

### EC-SOD Reduces Cardiac Fibrosis Through Ameliorating DNA Methylation

To elucidate the mechanism of how EC-SOD reduces cardiac fibrosis, we show that mRNA expression levels of DNA methyltransferases and methyl-CpG–binding domain proteins (MBD) were studied to investigate the possible mechanism for the observed methylation differences. Evaluated by RT-PCR normalized to GAPDH, DNMT expression was significantly higher in hypoxic WT than hypoxic TG cardiac cells (Figures 5A,B). The methylation level of the RASSFA1 promotor region (8 amplicons) was significantly higher in the hypoxic WT group vs. hypoxic TG group (Figures 8C,D). This suggests that the methylation pattern of the RASSFA1 promoter region may be attributed to differences in methyl-transferase expression and/or methyl binding proteins at mRNA level.

## Discussion

Free radicals play a key role in the pathogenesis of cardiac fibrosis [7]. Previously, it has been reported that the free radical scavenger, EC-SOD, can reduce, as well as reverse, some of the changes seen secondary to chronic hypoxic stress (19, 24). In our study, we reported that EC-SOD overexpression leads to a significant decrease in DNA methylation (DNMT1 and DNMT-3b as well as RASSFA1 gene), which is triggered by hypoxia exposure. Among animals subjected to chronic hypoxia, markers of fibrosis were increased significantly including RASSFA1 promoter methylation and SNAIL which is involved in fibro-modulation. Each of these changes was significantly reversed when EC-SOD was overexpressed in either *in-vitro or in-vivo model*. There was a significant increase in collagen 1, Collagen 3, and ASMA in hypoxic WT animals as compared to animals housed in room air (*P* < 0.05) (Figures 1A–C, 2A–C). Similar data was shown previously using human cardiac tissue [2], and it was shown that the degree of hypoxia was associated with increased expression of Collagen 1 and ASMA. In our animal model, overexpression of EC-SOD offered a significant protective effect, evident by reduction in the above listed fibrotic markers. Our data supports the role of oxidative insult induced by hypoxia, in the pathogenesis of cardiac fibrosis. The dismutation of these free radicals, by overexpression of EC-SOD, leads to reversal of heart pathology as shown by the immunochemistry studies (Figures 3A,B).

Many studies showed the role of SOD and its protective role in animal models with cardiac fibrosis induced by chronic hypoxia, by scavenging free radicals. SOD1 suppressed MCF proliferation and differentiation and reduced the production of collagen type I and III. SOD1 overexpression leads to ROS scavenging and blocking production and block collagen production, suggesting that SOD1 may be a promising therapeutic agent for treating ROS-mediated cardiac fibrosis (25). Mice deficient in SOD2 die of cardiomyopathy within 10 days of birth, whereas heterozygous SOD2(+/–) mice show ultrastructural damage of the myocardium and mitochondria, associated with an increased oxidative stress as well as an activation of apoptotic signaling pathways in the heart (26, 27). MnSOD overexpression offers protection against oxidative stress, fibrosis, and apoptosis in the aging heart (28). Serum EC-SOD activity was independently associated with abnormal LV geometry patterns with and without overt HF. Our results indicate that Ec-SOD might be a potential link between LV structure remodeling and the development of subsequent HF in patients with cardiovascular disease. Extracellular superoxide dismutase is associated with left ventricular geometry and heart failure in patients with cardiovascular disease (29). Under unstressed conditions, EC-SOD deficiency had no effect on myocardial total SOD activity but resulted in small but significant increases in myocardial fibrosis and ventricular mass. EC-SOD deficiency is associated with exacerbated myocardial oxidative stress, hypertrophy, fibrosis, and dysfunction. All these findings indicate that the distribution of EC-SOD in the extracellular space is critically important in protecting the heart against pressure overload (17). In our study, we report an innovative mechanism of overexpression of EC-SOD. In animal models with cardiac fibrosis induced by chronic hypoxia, there is a significant increase of ERK1/2 in the hypoxic animal group in parallel with a significant increase of DNA methylation and a reduction of RASSF1A expression. EC-SOD overexpression reverses this process and leads to significant decreases in DNA methylation (DNMT1 and DNMT-3b as well as RASSFA1 gene), which is triggered by chronic hypoxia exposure.

The role of DNA methylation and epigenetic changes associated with cardiac fibrosis induced by hypoxia was studied previously (15, 18). Both DNA methyltransferase enzymes (DNMT1 and DNMT3b), which are regulated by HIF-1α (30) (Figure 5C), are upregulated by chronic hypoxia (Figures 5A,B). Their upregulation was associated with increases in all fibrotic markers examined and a significant reduction in RASSF1A protein synthesis (Figure 6). In addition, the significant increase in the expression of both DNMT enzymes was associated with increased DNA methylation (Figure 6). Epigenetic changes induced by prolonged hypoxia have been shown to contribute to the pro-fibrotic nature of the ischemic environment (2). In normal human lung fibroblasts, there is a significant global hypermethylation detected in hypoxic fibroblasts relative to normoxic controls and is accompanied by increased expression of myofibroblast markers (24).

SNAIL gene expression is a potential target molecule in cardiac fibrosis after ischemia reperfusion (I/R), injury, and or oxidative stress insult (31). The SNAIL gene is best known for its capability to trigger epithelial-to-mesenchymal transition (EMT) and endothelial to mesenchymal transition (EndMT), which may contribute to myofibroblast formation (30, 32). SNAIL is activated by free radicals and mediates the actions of endogenous TGFβ signals that induce EndMT (33). Injection of a selective SNAIL inhibitor remarkably suppressed collagen deposition and cardiac fibrosis in mouse I/R injury and significantly improved cardiac function and reduced SNAIL expression *in-vivo* (34). SNAIL can recruit multiple chromatin enzymes including LSD1, HDAC1/2, and Suv39H1. These enzymes function in a highly organized manner to generate heterochromatin and promote DNA methyltransferase-mediated DNA methylation at the promoter region (35). Our data showed that a significant increase of SNAIL expression in the WT hypoxic group was attenuated in TG animals, which may lead to a disruption of the connection between SNAIL and these chromatin-modifying enzymes and may represent a therapeutic target for the treatment of cardiac fibrosis.

Hypoxia-induced DNA methylation has been shown to be involved in regulating the process of cardiac fibrosis (2, 35). DNA methylation mediated silencing of the RASSF1A gene, which leads to upregulation of ERK1/2 that has been shown to increase cardiac fibrosis in cancer patients under chronic hypoxic stress (6, 36). Our data showed a significant increase of ERK1/2 in the hypoxic animal group in parallel with a significant increase of DNA methylation and a reduction of RASSF1A expression (Figure 6A). In adult cardiomyocytes, the high level of H_2_O_2_ is associated with the activation of ERK1/2 MAPK and the stimulation of protein synthesis (37). Increased ERK1/2 activity leads to increased cell proliferation and collagen gene expression in activated cardiac fibroblasts [4]. Dismutation of the free radicals by the activity of EC-SOD leads to a global decrease of DNA methylation, increased RASSF1A protein synthesis, and a significant reduction in phosphorylated ERK1/2 in the transgenic hypoxic animal group (*P* < 0.05). This finding suggests an additional contributing mechanism to cardiac dysfunction in hypoxia, which is triggered by a change in myosin heavy chain isoform (38). Previously, it has been shown that hypoxia leads to a change in MHC from the α to β isoform, which leads to decreased cardiac contractility (37). Free radicals have been shown to affect this change and scavenging these free radicals by antioxidants can markedly attenuate cardiac fibrosis and improve ventricular ejection fraction and fractional shortening (38). In our study, there was a significant reduction in the levels of α-MHC (Figure 7A) and significant increase in b-MHC (Figure 7B), in hypoxic WT animals as compared to the hypoxia TG group as shown in the RT-PCT assay. Hypoxia TG animals had both α&β-MHC levels close to RA controls. This critical histological change is crucial in preserving cardiac contractility and function.

We have shown that TG animals that have an additional copy of the EC-SOD gene show a significant reduction in DNA methylation in response to chronic hypoxic stress. While further studies are needed to completely clarify the mechanism, we speculate that this reduction in DNA methylation is through a reduction in the levels of HIF-1α, which is an inducer of DNMT and hence of the process of DNA methylation (6). Our data show a significant reduction in HIF-1α levels in the animals that overexpress EC-SOD (*P* < 0.05) (Figure 5C). This suggests a mechanism by which DNA methylation leads to cardiac fibrosis. Both these changes were mitigated in our transgenic animals, which overexpress EC-SOD.

To further explore the role of RASSF1A in cardiac fibrosis, its expression was blocked in an *in-vitro* model using SiRNA. The presence of the SiRNA resulted in a significant increase in fibroblast proliferation (Figures 8A,B). Furthermore, we investigated RASSF1A promoter gene methylation in the three studied groups. Our findings showed a significant increase in RASSF1A promotor region methylation in the hypoxic WT group compared to normoxic animals. This methylation process was reduced by more than 10% among the hypoxic TG animals, which showed a significant reduction of both biochemical and histopathological evidence of cardiac fibrosis.

Epigenetic inactivation of RASSF1A by methylation is a very common event in prostate cancer and might be involved in the progression of the disease (39). In prostate cancer, hypomehtylation of BNIP3 and hypermethylation of both EC-SOD and RASSF1A were observed. These changes were positively associated with oxidative stress and inverse associated with *EC*-*SOD* expression. Among patients with prostate cancer, it was found that glutamate carboxypeptidase II genetic variants contribute to increased oxidative stress and prostate cancer risk by modulating the CpG island methylation of Ec-SOD (40).

In summary, our study presents a novel mechanism by which EC-SOD offers cardiac protection against fibrosis induced by chronic or prolonged hypoxia. The data identifies a critical role of EC-SOD in the control of DNA methylation. Our proposed mechanism, illustrated in Figure 8E, suggests that EC-SOD expression will chelate the free radicals, induced by hypoxia; as a result, both HIF-1α and SNAIL gene activation will be decreased and subsequently methylation enzyme activity will decrease and RASSF1A gene expression will not be silenced or decreased due to lack of methylation. RASSF1A expression downregulates the activity of the ERK1/2 pathway which regulates activation of both cardiac fibroblast proliferation and transition of endothelial cells to myofibroblast. Another benefit from using antioxidants is chelating hydrogen peroxide, which will significantly decrease the activation of ERK1/2, as it is triggered directly by hydrogen peroxide concentration. Previously EC-SOD compounds and its mimetics were used in experimental and clinical trials to counteract the oxidative stress which was linked to the pathogenesis of these disorders (41–46). Further studies of this mechanism could lead to specific inhibition of the pathway in the clinic to significantly reduce cardiac fibrosis and dramatically improve the outcome of this devastating condition.

## Data Availability Statement

The original contributions presented in the study are included in the article/Supplementary Material, further inquiries can be directed to the corresponding author/s.

## Ethics Statement

The animal study was reviewed and approved by Institutional Animal Care and use Committee of the Feinstein Institute for Medical Research. Written informed consent was obtained from the owners for the participation of their animals in this study.

## Author Contributions

MA and AR designed the research. KA, JL, NZ, and AR performed the research and analyzed the data. EM and MA wrote the manuscript. All authors contributed to the article and approved the submitted version.

## Conflict of Interest

The authors declare that the research was conducted in the absence of any commercial or financial relationships that could be construed as a potential conflict of interest.

## Supplementary Material

Supplementary #1 Strategy for RASSF1A sequencing.

Supplementary #2 Methylation study design.

Supplementary #3 Comparison between RA WT control group and RA TG groups. **(A)** Collagen 1: Comparison between RA WT and RA TG groups, no significant difference (*P* <0.05). **(B)** ASMA: Comparison between RA WT and RA TG groups, no significant difference (*P* < 0.05). **(C)** SNAIL1: Comparison between RA WT and RA TG groups, no significant difference (*P* < 0.05). **(D)** DNMT1: Comparison between RA WT and RA TG groups, no significant difference (*P* < 0.05). **(E)** DNMT 3B: Comparison between RA WT and RA TG groups, no significant difference (*P* < 0.05). **(F)** HIF-1α&β Comparison between RA WT and RA TG groups, no significant difference (*P* < 0.05). **(G)** RASSF1A: Comparison between RA WT and RA TG groups, no significant difference (*P* < 0.05). **(H)** Phosphorylated ERK $\frac{1}{2}$: Comparison between RA WT and RA TG groups, no significant difference (*P* < 0.05).

## Abbreviations

RASSF1A: Ras association domain family 1 isoform A
ERK1/2: Extracellular signal regulated kinases
EC-SOD: Extracellular superoxide dismutase
ROS: reactive oxygen species
WT: Wild type
TG: Transgenic
DNMT: DNA methyltransferase
SNAIL: Zinc finger protein SNAI1
MHC: Myosin Heavy chains
Col: Collagen
ASMA: Alpha smooth muscle actin.
