# Supplementary material for: Extracellular Superoxide Dismutase (EC-SOD) Regulates Gene Methylation and Cardiac Fibrosis During Chronic Hypoxic Stress
Source: Front Cardiovasc Med. 2021 May 31;8:669975. doi: 10.3389/fcvm.2021.669975 (PMC8202000; doi:10.3389/fcvm.2021.669975)

Supplement # 1

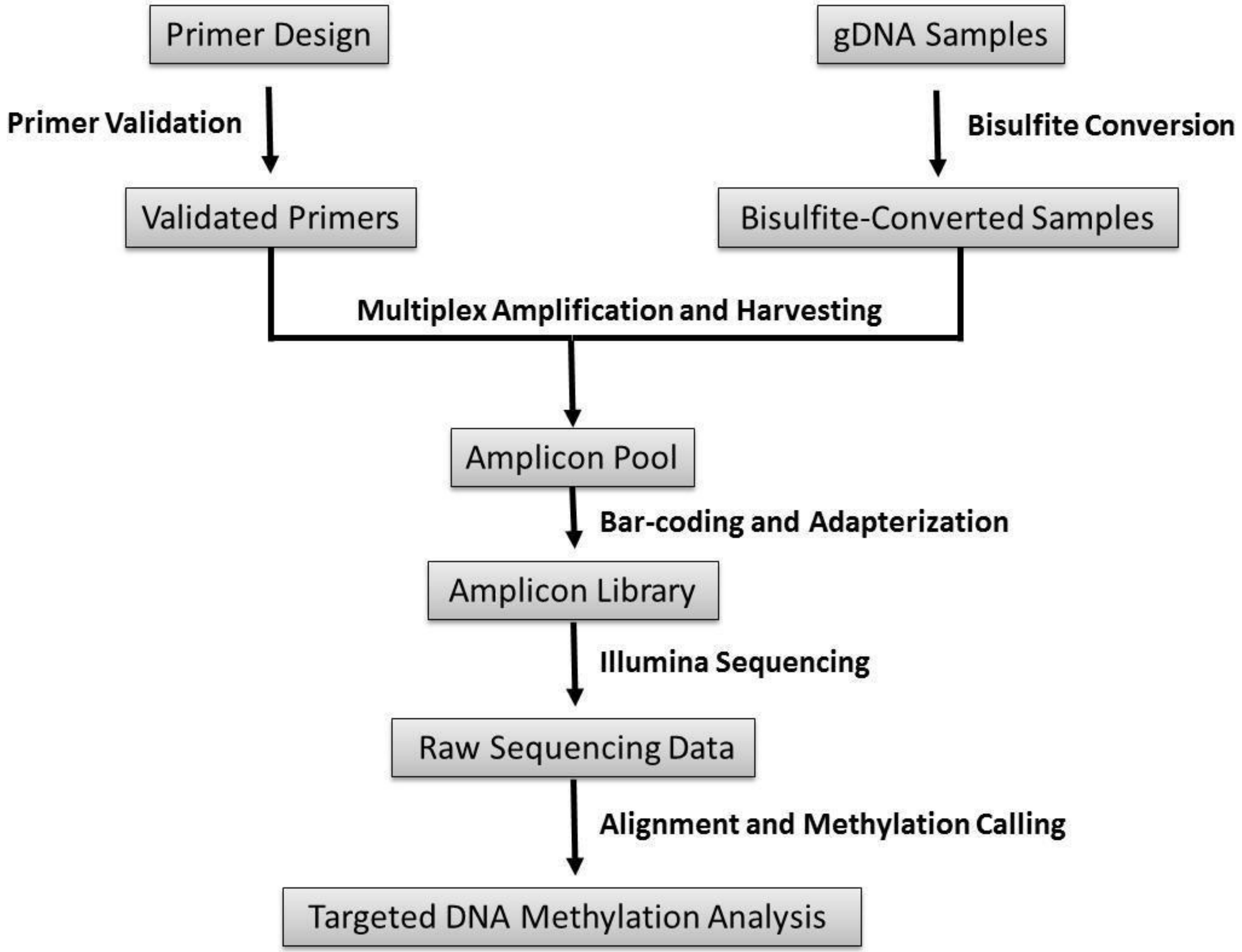

Supplement # 2

```
CGGCATCCGGTGGGCTCCTTCTCCTCCCCCAGGCAAGCTGCAGCATTTTGAGAGTGGCCGATGGCAGACGGTGGC
CCCCTCAGAGCAGCAAAAGCTGAATAAACTGGATGGGCAAGTATGGATCGCCCTGTACAATCTACTGCTCAGCCC
TGAGGCCCGAGCCCGTTACTGCCTTACAAGCTTTGCCAAGGGACAGCTGCTTAAGGTGGAGAACTCAATGACCCC
GCCCAGCCCCACTGCCCCCCTGCCCAGACCCACCCCCCAGGGCATCCTGGCCTGTCCCTGTCCCCTTTCCACAAC
TTGGGCTTCAGGCATCGACAGGGGAGCTGGTAAGATGCAAACCCTCCAAGGACGCTCAGCCCAATTTCAGGACTT
CCTGTCTCTTGGCCCTCTGGCTAGGAACAGTTAGTTTCTCAATGGCCTTCCCTGGATCTCTTGCAGCTTCAGGCC
TTCCTCACTGACACACTACTCGACCAGTTGCCCAATCTTGCCGATCTGAAGGGTTTCCTGGCCCACCTGTCCCTG
GCTGAAACCCAGCCCCCTAAGAAGGACCTAGTGTTAGAACAGGTAAGGCCCTAGAACATGCTTGGGAGTGCTTGG
GACGGTGGTCCACTTTTCCGGCTCCTTCCTCCCTGTCTCCATTACACTTTTTTAGATCCCAGAAATCTGGGATCG
CCTGGAGAGAGAGAACAAAGGGAAATGGCAGGCTATCGCCAAGCACCAGCTTCAGCACGTATTCAGCCTCTCGGA
GAAGGATCTTCGTCAACAAGCACAGAGGTAAAGTCCACTGAGTGGCTGAAGTCACGTGCATAGGACCAAGGAGGG
AAGGTCACCTAGTGCTTCTGTGCACCTACCTGCCCGGTCCGTCCATTCAGCTCTTCTCGACTCCTCCCCCAGGTG
GGCTGAAACCTACAGGCTGGATGTCCTAGAGGCAGTAGCTCCGGAGAGGCCCCGCTGCGGCTACTGCAACGCAGA
GGCCTCCAAGCGCTGCTCCAGATGCCAGAATGTGTGGTATTGCTGCAGGTGAGGCGATCCTGGGACCTTAGTTGC
CCCCCTAAGCTCCAACCCTGGGTTCTCCTACAACCCCTGTCATTGTCATGCAACCCACCCCGCTGCAGGGAGTGT
CAAGTCAAGCACTGGGAGAAGCACGGAAAGACATGTGTTCTAGCAGCCCAAGGTGACAGAGCCAAGTGAAGGCCG
TAGCTGCCGAGGGCCGACAGAAGAGCGCACCCAGACTGTGACTGAGCTTCAGGGACTCGTGGCTCGCCCCTGTCA
GACCTCAATTTCCCTGGTGAGCACAGCTGAGTCAAGTAGAGCTGCAAACCACCACACTCTTCACTTCCTGCCCCA
CCCAGCGAATACGCAGAGGACCAGGCCACAGCATCAGCAAACTGGCAGACAGGGTTGGGGGTGTAAGCAAGGGC
CGGATGTGGAGACCCTCTTCCTTTCGCACAATAAAGCAGACTCATGAAACGCAACAGTGTGGCGCTGTACAGTCA
TCCTTGGGGTCGGGGGAGAAGACTGGACGGAAAGATTGGGAAGGGGCTCTTTGAAAGGGCCCACCCCTGTGCCGT
GCCCCGCCCACAAGTCCACACGGTCGGCTCGCCTAGGGTTCCAGCGCGTCCTCCTCGGCTCTTTCCCGCCGCCCT
GCCTGGATCCTGGGGGCGGTGCCGAAGTCGCGGCCCGCCCTGCGGCCTCGCCCGCTCGGTACTCACTAGCTGCTA
GTCCGCTACAGCACCGGCCTGACCGGCCATGTCGGCGGAGCCAGAACTCATTGAACTACGCGAGCTGGCACCGT
CCGGGCGCATCGGTCCAGGCCGTACCCGGCTGGAGCGTGCCAACGCTCTGCGCATCGCCCCGGGTACAACACGCA
ATCCGTCACAGCAGCACGTCCCGGGTCGTGGCCACCGTTTCCAGCCTGCAGGGCCCACCACGCACACGTGGTGCG
ACCTCTGTGGAGACTTCATCTGGGGCGTCGTGCGCAAGGGCCTACAGTGC
```

Small Letters = Repeat Sequences; Capital Letters = Unique Sequence; Dark Gray= Regions not covered by Targeted Sequencing Amplicons, Light Gray= Regions of Interest; CG= CG of interest >mm10. DNA range=chr9:107549843-107551842 5'pad=0 3'pad=0 Estimated No. of amplicons: 8.

Supplement # 3A

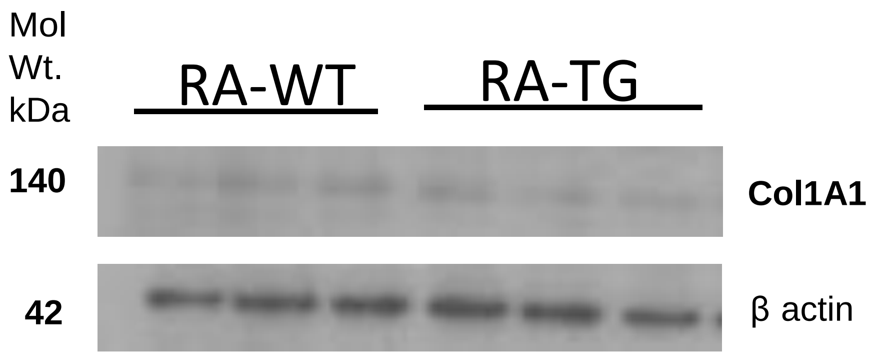

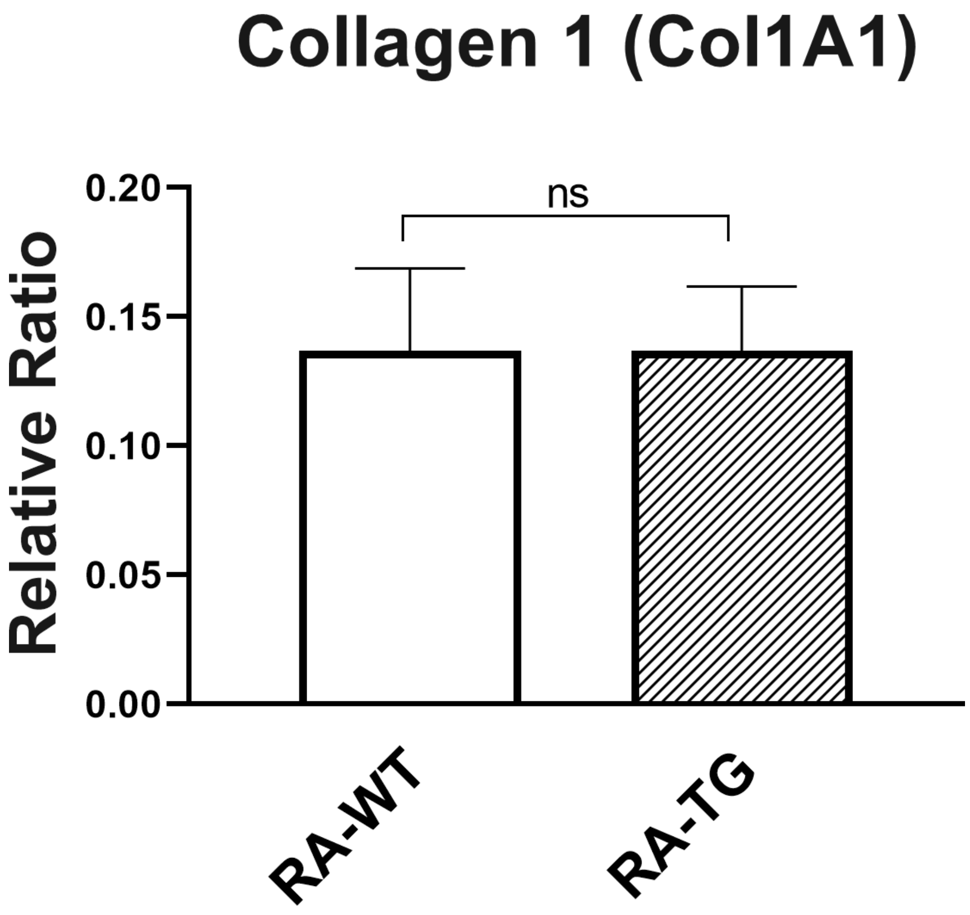

Supplement # 3B

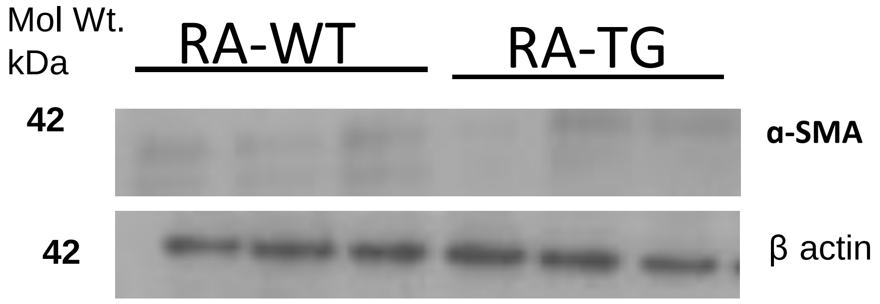

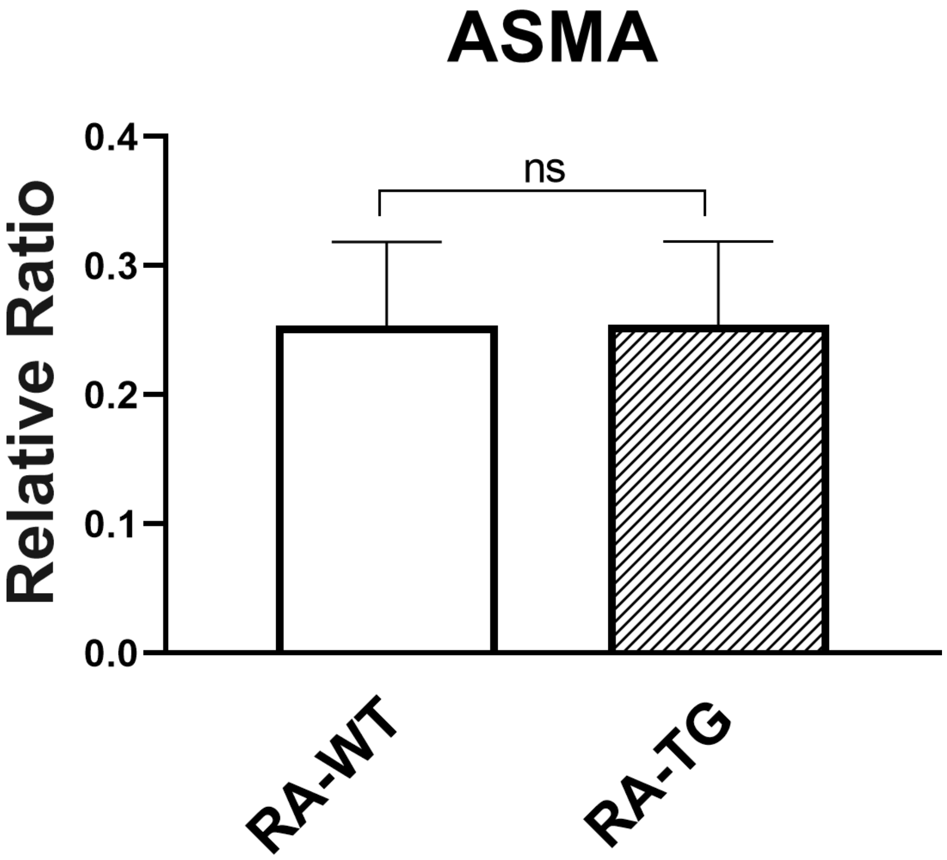

Supplement # 3C

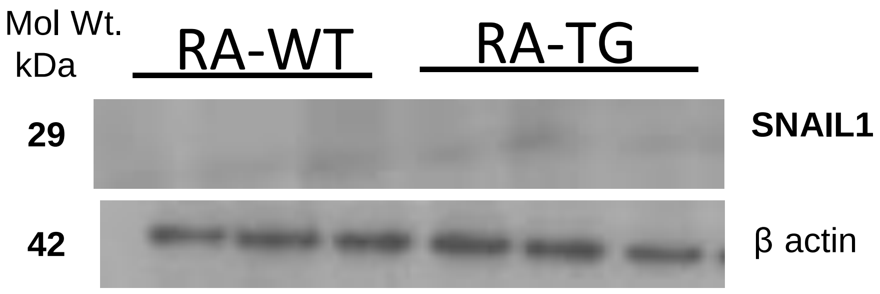

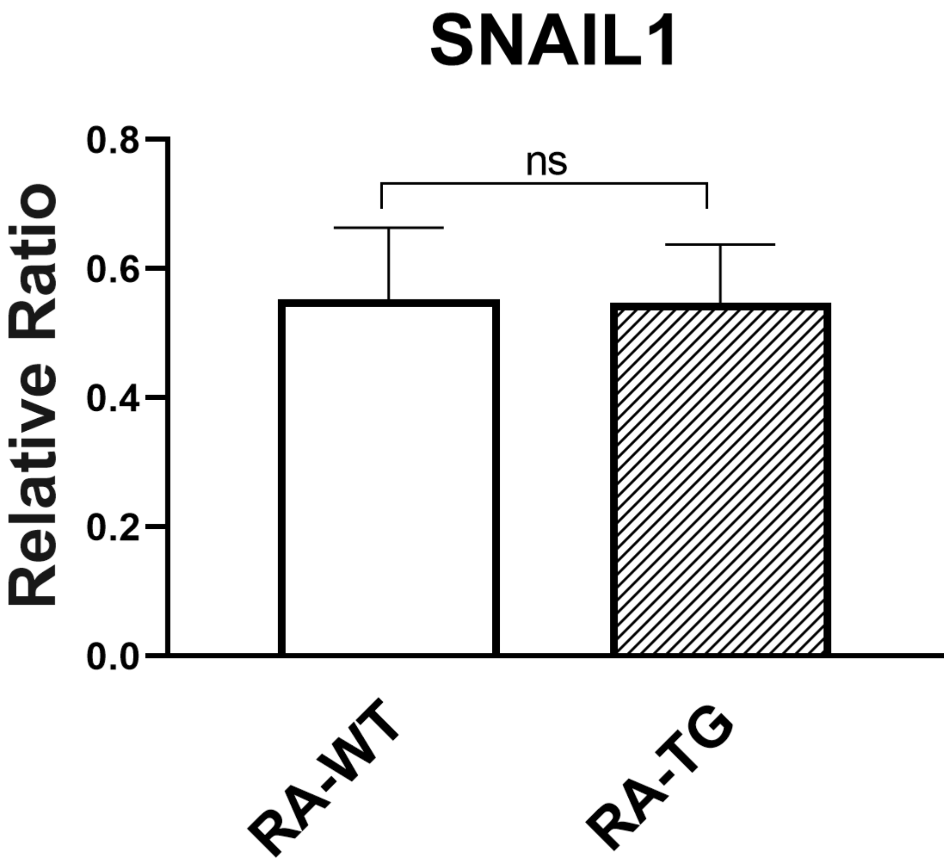

Supplement # 3D

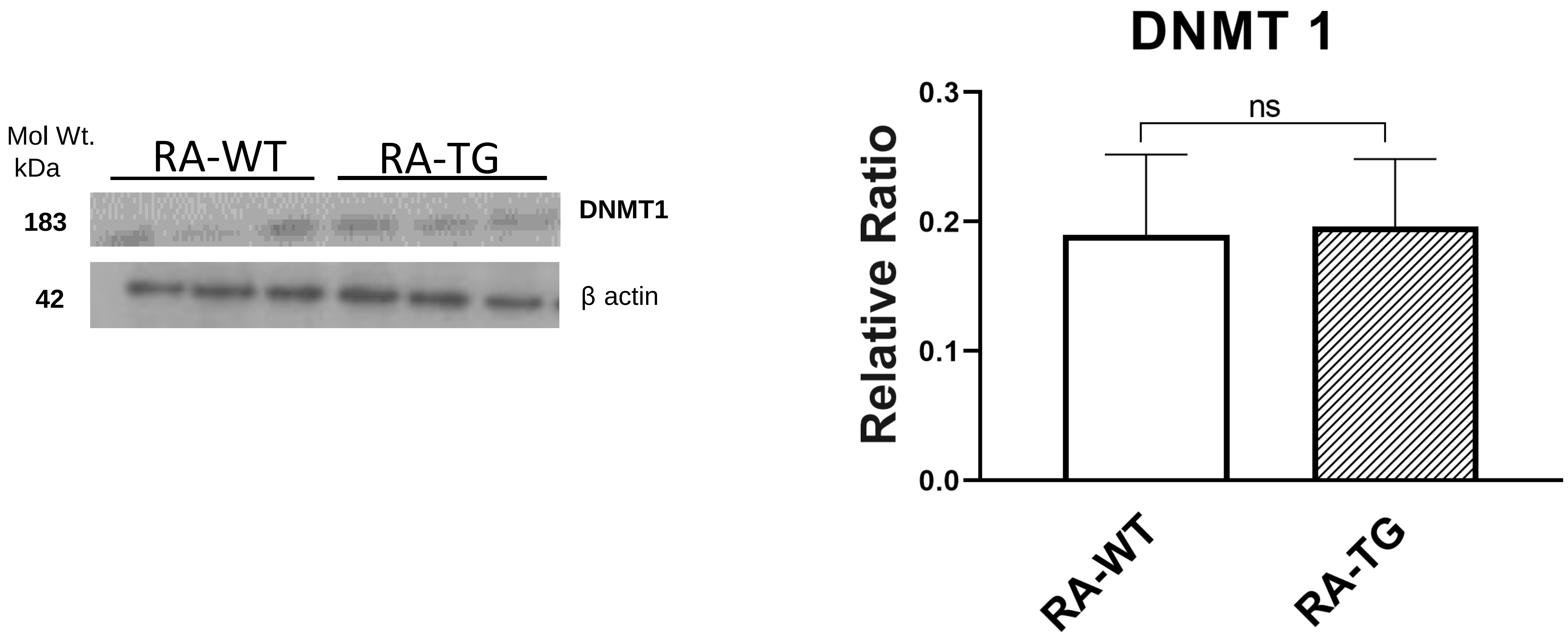

Supplement # 3E

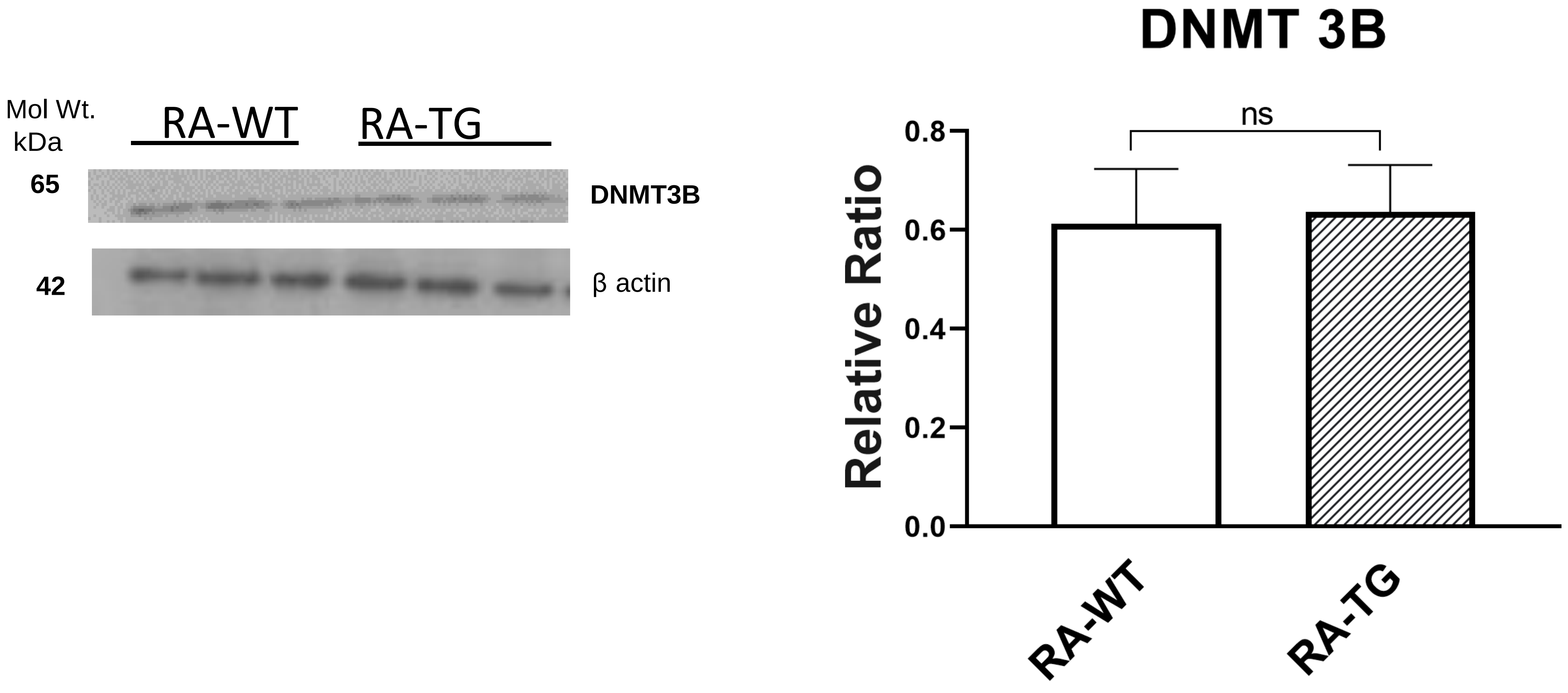

Supplement # 3F

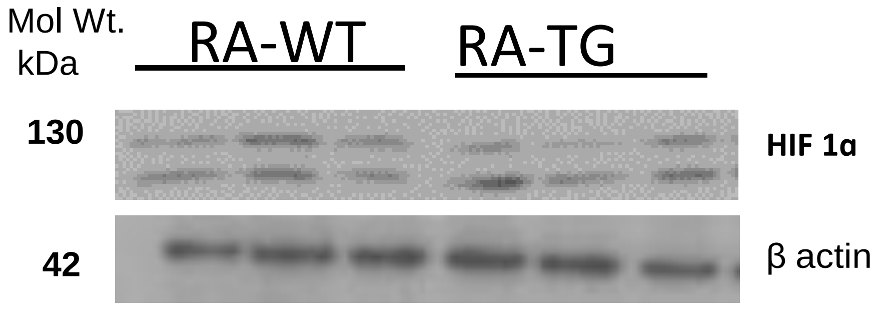

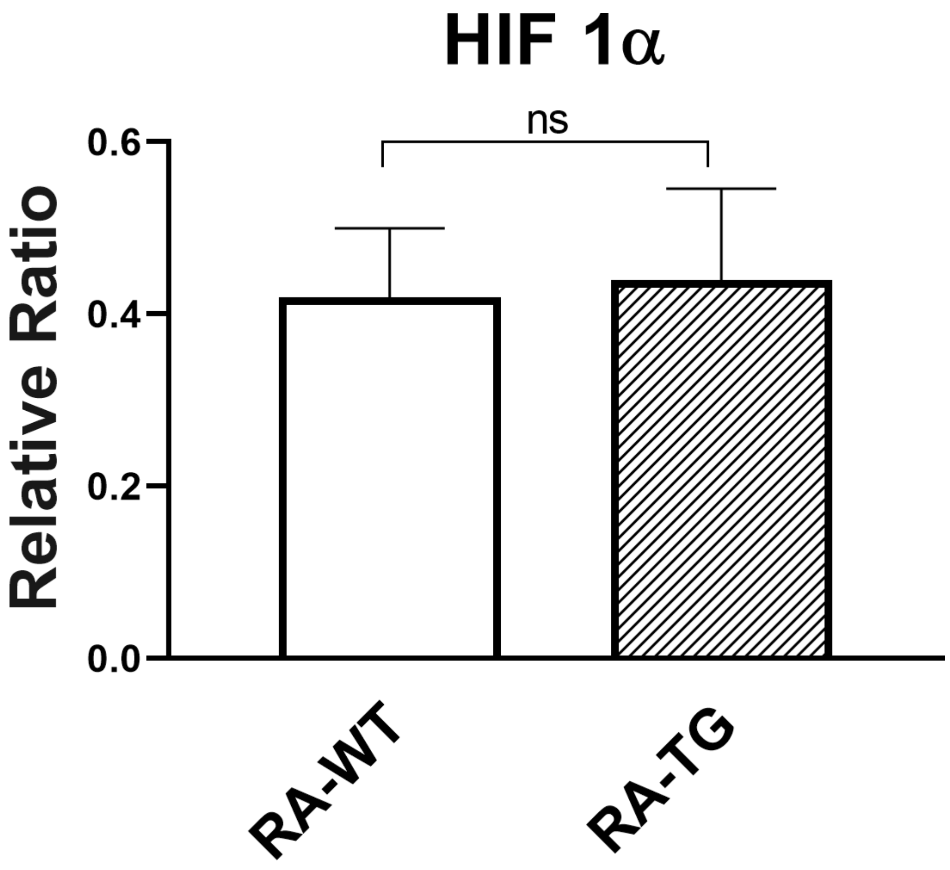

Supplement # 3G

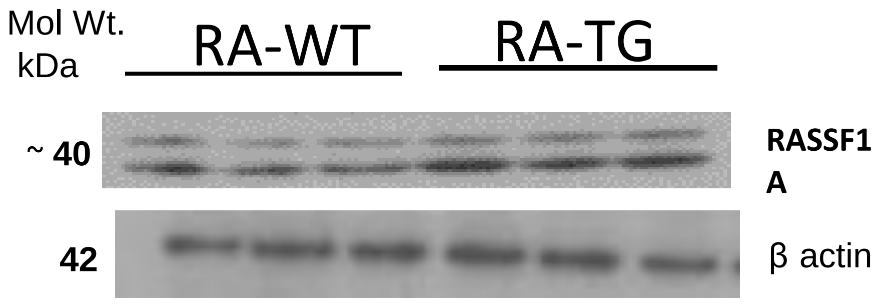

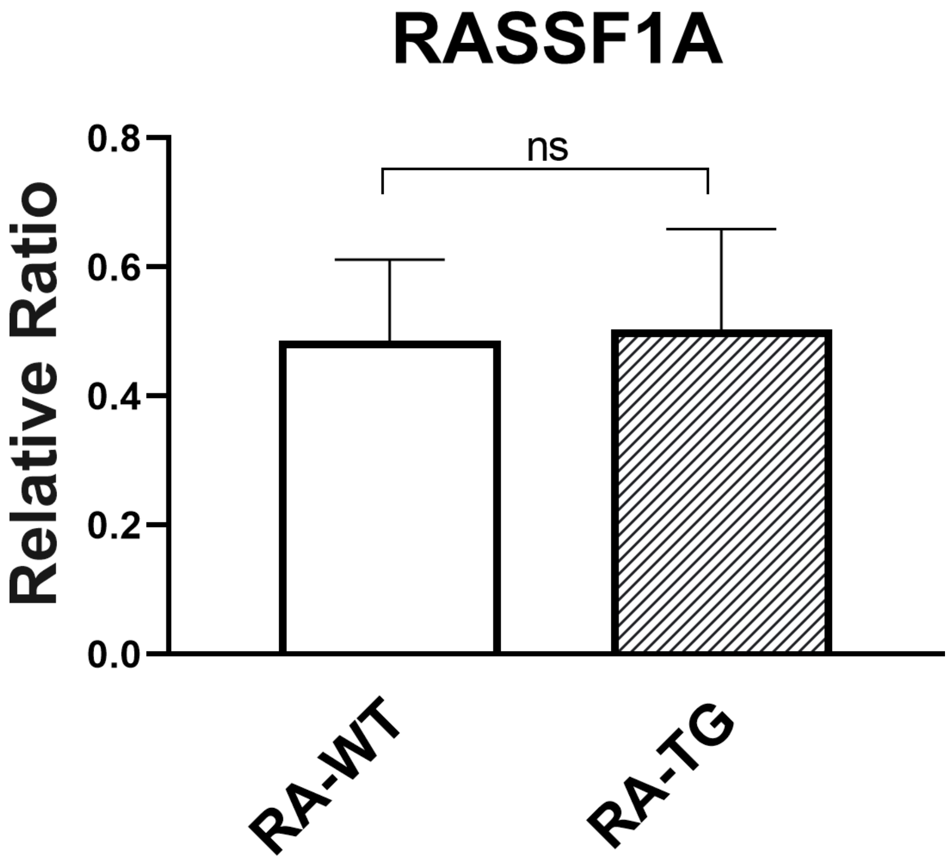

Supplement # 3H

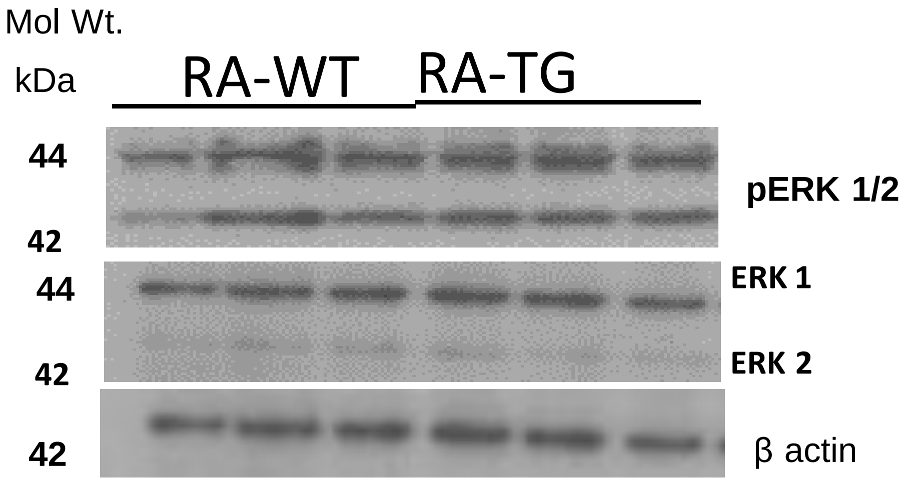

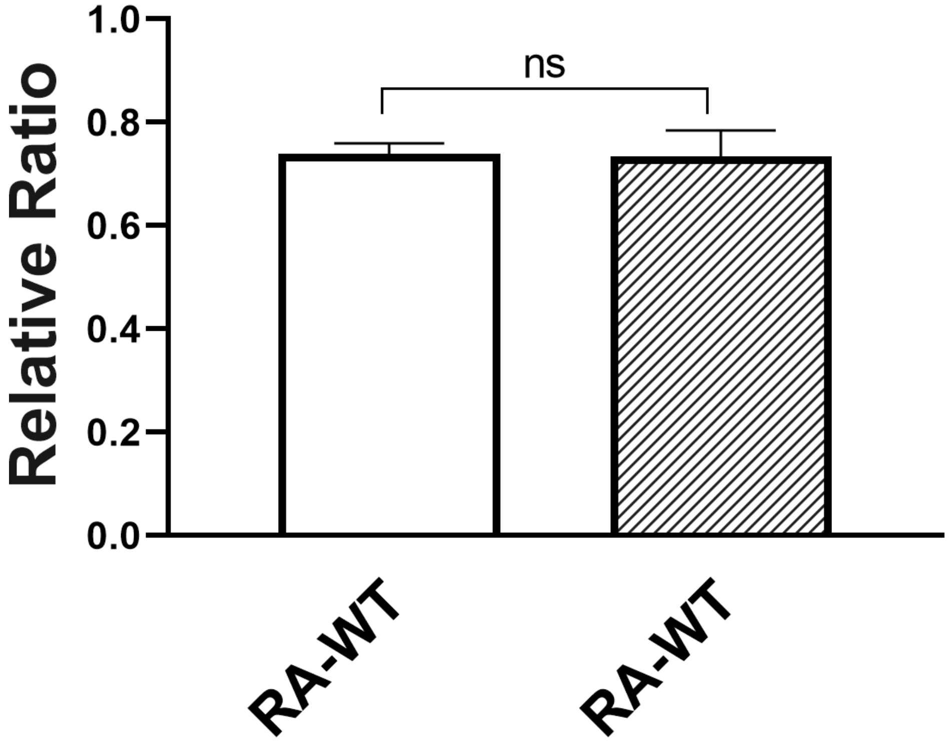

Supplement: Supplementary #1 — Strategy for RASSF1A sequencing. [file Data_Sheet_1.pdf]
